# Neural correlates of nesting behavior in zebra finches (*Taeniopygia guttata*)

**DOI:** 10.1016/j.bbr.2014.01.043

**Published:** 2014-05-01

**Authors:** Zachary J. Hall, Marion Bertin, Ida E. Bailey, Simone L. Meddle, Susan D. Healy

**Affiliations:** a School of Biology, University of St. Andrews, Harold Mitchell Building, St. Andrews, KY16 9TH Scotland, United Kingdom; b The Roslin Institute, The Royal (Dick) School of Veterinary Studies, The University of Edinburgh, Easter Bush, EH25 9RG Scotland, United Kingdom

**Keywords:** AH, anterior hypothalamus, ASt, anterior striatum, AMV, anterior ventral mesopallium, AN, anterior nidopallium, BSTl, bed nucleus of the stria terminalis, lateral subdivision, BSTmd, bed nucleus of the stria terminalis, dorsomedial subdivision, BSTmv, bed nucleus of the stria terminalis, ventromedial subdivision, dHP, dorsal hippocampus, DLN, dorsolateral nidopallium, GCt, central gray, LAI, lateral intermediate arcopallium, LScv, lateral septum, ventral caudal subdivision, LScvl, lateral septum, lateral ventral caudal subdivision, LSr, lateral septum, rostral subdivision, mHP, medial hippocampus, MS, medial septum, NIML, nidopallium intermedium medialis pars laterale, PBS, phosphate-buffered saline, POM, medial preoptic area, RA, robust nucleus of the arcopallium, TnA, nucleus taeniae, VMH, ventromedial hypothalamus, VTA, ventral tegmental area, Nesting behavior, Nest building, C-fos, anterior motor pathway, Zebra finch

## Abstract

Nest building in birds involves a behavioral sequence (nest material collection and deposition in the nest) that offers a unique model for addressing how the brain sequences motor actions. In this study, we identified brain regions involved in nesting behavior in male and female zebra finches (*Taeniopygia guttata*). We used Fos immunohistochemistry to quantify production of the immediate early gene protein product Fos (a molecular indicator of neuronal activity) in the brain correlated this expression with the variation in nesting behavior. Using this technique, we found that neural circuitry involved in motor sequencing, social behavior, reward and motivation were active during nesting. Within pairs of nesting birds, the number of times a male picked up or deposited nesting material and the amount of time a female spent in the nest explained the variation in Fos expression in the anterior motor pathway, social behavior network, and reward neural circuits. Identification of the brain regions that are involved in nesting enables us to begin studying the roles of motor sequencing, context, and reward in construction behavior at the neural level.

## Highlights

• We compare markers of neural activity to nesting behavior in zebra finches.
• We visualized immediate early gene (Fos) expression in nesting and control finches.
• Fos production in motor, social, and reward neural circuits correlated with nesting.
• Fos production correlated with material pick-up in male nesting finches.
• Fos production correlated with time spent in the nest in female nesting finches.

## 1 Introduction

Nest building in birds consists of a sequence of motor actions, which in its simplest form involves the collection and deposition of nesting material. For some species nest building can be decomposed into just a few actions while for others the construction of some nests is more elaborate. For example, arctic terns (*Sterna paradisaea*) nest in unadorned ground scrapes whereas long-tailed tits (*Aegithalos caudatus*) sequence up to 14 motor actions to construct their domed nest of moss and spider egg cocoons [1]. Superficially at least, nest building appears to involve motor actions and sequencing akin to those used in tool manufacture and use [2–5] but to date there is little information regarding the neural underpinnings of these behaviors in birds.

In this study, we sought to investigate the neurobiology of nest building in zebra finches (*Taeniopygia guttata*). Zebra finches readily construct nests in the laboratory [6–8] using an easily-quantified motor sequence of nest material collection and deposition. While the male zebra finch collects and deposits nest material, the female manipulates material to shape a species-typical dome nest [9]. To identify brain regions involved in nesting behavior, we quantified the production of the immediate early gene c-*fos* protein product Fos (a molecular indicator of neuronal activity; e.g. [10,11]) throughout the brain in male and female zebra finches that did or did not construct a nest. We quantified Fos immunoreactivity in the anterior motor pathway, which is thought to control motor learning and sequencing [12] and includes the striatum, the input structure of the basal ganglia. The basal ganglia control motor planning and sequencing in vertebrates [13] and are activated during trained tool use in macaque monkeys [14].

In this study, we tested the hypothesis that nest building involves motor planning and predicted that Fos immunoreactivity in the anterior motor pathway would correlate with nest-building behavior in male zebra finches. We also predicted that Fos expression would not differ between nest-building and control birds (birds that were not allowed to build nests) in the posterior motor pathway, a circuit that is involved in the production of motor actions [12], as both nesting and control birds could move freely.

As the social behavior network contains brain regions involved in avian reproductive and parental behavior (e.g. [15]) we quantified Fos expression in those regions. Based on the recent demonstration of the involvement of vasotinergic neural circuitry in female zebra finch nesting behavior [16] and that nest box possession in starlings increases Fos expression in the social behavior network [17], we predicted that Fos immunoreactivity specifically in BSTmd, BSTmv, AH, POM, and VMH would be higher in nesting birds relative to control birds. While Heimovics and Riters [17] noted that starlings that possessed a nest box also constructed nests they did not quantify nesting behavior and so were unable to test whether nest building was associated with Fos production in the social behavior network. By quantifying nest-building behavior, we could test whether Fos production in these regions during nest building is associated with nest possession or nest building itself.

Based on the assumption that nesting is a rewarding behavior we predicted that Fos expression in the dopaminergic reward circuit, which is involved in reward and motivation of motor behavior [18], would correlate with nesting behavior. We expected to see this correlation specifically in VTA and GCt, two dopaminergic reward nuclei in which nest box possession in starlings leads to increased Fos immunoreactivity [19,20].

Lastly, as the avian hippocampus is implicated in spatial memory and in synthesizing multimodal cues, we tested whether the hippocampus was involved in initiating nest building after recognizing a reproductive context [21,22].

## 2 Methods and materials

### 2.1 Animals

Thirty-two adult zebra finches (*Taeniopygia guttata*; *n* = 16 male, *n* = 16 female) were bred in captivity at the University of St. Andrews, St. Andrews, Scotland, UK and the University of Glasgow, Glasgow, Scotland, UK. All of the males had previously built nests using coconut fiber [6]. Prior to experimentation, birds were housed in single sex groups in cages containing 10–20 birds with access to finch seed mix and water *ad libitum* but deprived of access to coconut fiber. The room was held on 14L:10D light:dark light cycle (lights on 8:00) with temperatures ranging between 19–27 °C and 50–70% humidity. All procedures were performed with ethical permission from the University of St Andrews Animal Welfare and Ethics Committee and from the UK Home Office (PPL. 60/3666).

### 2.2 Treatment group assignment

In preparation for the experiment, zebra finches were caught from group cages and randomly paired (one bird of each sex) in wooden/wire mesh cages (44 × 30 × 39 cm), which were then moved to a separate room (holding only paired finches) with the same light cycle, temperature, and humidity as the group-housing room. The cages were fitted with a wooden nesting cup (11 × 13 × 12 cm) and the floor was covered with wooden bedding chips. The birds had access to finch seed mix and water *ad libitum*. Birds were paired for at least one week before they were provided with coconut fiber as nesting material. Prior to receiving nesting material, all pairs filled their nest cup with bedding chips from the cage floor at least once and some females laid eggs in these nests. All bedding and eggs were removed from nest cups after daily inspection.

At least one week after pairing, six pairs of birds were given 7.5 g of coconut fiber at 12:00 (4 h after lights on). We inspected cages on the following day at 12:00 to identify pairs that had begun depositing material in the nest cup. To create an experimental cohort, we randomly assigned a pair of finches that had begun building a fiber nest to each behavioral treatment group (nesting or non-nesting control group). To ensure that all of the finches included in this study were motivated and capable of building nests prior to behavioral observation for both nesting and control groups we selected only pairs of birds that had begun nest building. We removed nests and remaining fiber from the cages of both pairs and the nest cup from the cage of the control pair. We also removed the cage bedding chips and lined the cage floor with black plastic to prevent unwanted nest building with bedding. The two pairs were then moved to an isolation room.

### 2.3 Isolation of nesting behavior

Once in the isolation room, the control and experimental pairs were visually but not acoustically isolated from each other by a wooden barrier.

On the next morning, 1 h after lights on, we gave the nesting pair 12 g of coconut fiber and monitored them throughout the day for evidence of nest building. If the nesting pair began constructing a nest within the day they received nesting material, we scheduled the behavioral observation period for the next morning. If the nesting pair failed to construct a nest on the first day we provided the material, we replaced the 12 g of coconut fiber the next morning and monitored the nesting male for the remainder of the day. If a nesting male failed to deposit any material in the nest cup within two days of material provision, the nest cup and material were removed and a new nest cup and 12 g of coconut fiber were given to the control pair, reversing the treatment assignment of each pair in the cohort. Reversal of treatment conditions occurred twice and in one case, neither male constructed a nest while in the isolation room. These birds were removed from the study and replaced by a subsequent cohort.

We removed unused nest material when the lights came on the morning after a nesting pair began nest building in the isolation room. Both pairs were left for 30 min before we began filming. After 30 min, we gave the nesting pair 9 g of coconut fiber so that the male could resume nest building and we filmed each pair using either a JVC Everio ACVHD (Model no. GZ-HD300AU) or Sony Handycam AVCHD (Model no. HDR-CX115E) camcorder. Nest-building males did not typically resume construction immediately so we observed the birds from outside the isolation room via a window until we observed the nesting male make three consecutive trips with material from the cage floor to the nest. We recorded the time at which the male began to build.

### 2.4 Behavior coding

We encoded the birds’ behavior using Noldus Observer (TrackSys Ltd., Nottingham, UK) behavioral analysis software. We measured the occurrence of five behaviors that were performed by all of the birds: *hopping* (a jump between perches, the cage floor, and/or the nesting cup), *feeding* (pecks into the ground or cage-mounted feeder), *drinking* (pecks into the cage-mounted water dispenser), *preening* (each preen of the chest, wing, or tail feathers by the beak), and *scratching* (bird lifts leg and scratches head feathers with foot). In females, we also recorded *allopreening* (each time the female preened her partner male with her beak). In males, we assessed singing behavior in two ways: *song bouts* (number of song bouts separated by at least 3 s) and *time spent singing* (number of seconds a bird spent singing). We measured two behaviors unique to the nesting males: *pick up* (male picked up coconut fiber from the floor of the cage using his beak) and *put down* (male released coconut fiber into the nest cup). In both nesting males and females, we counted the number of *nest visits* (bird entered the nest cup) and *nest time* (number of seconds the bird spent in nest cup).

### 2.5 Tissue collection

After 90 min following the initiation of nest building, an experimenter entered the room to confirm visually that material on the floor of the cage had been added to the nest. Once confirmed, both pairs of control and experimental birds were terminally anaesthetized (0.2 ml Pentobarbitone sodium i.p.; Dolethal, Vétoquinol) and brains were rapidly dissected from the skull. Brains were fixed via submersion in 4% paraformaldehyde in PBS (0.1 M, pH 7.4) for six days and cryoprotected in 20% sucrose in PBS for 48 h. The brains were then embedded in quail egg yolk, which was subsequently fixed with 4% paraformaldehyde over six days. The embedded brains were sectioned coronally (section thickness = 30 μm) using a freezing microtome and sections were collected in three, alternating series (intersection interval = 90 μm) into 0.1 M PBS.

We repeated all of these procedures until we had observed behavior of, and collected brains from, eight nesting pairs and eight control zebra finch pairs.

### 2.6 Fos immunohistochemistry

We rinsed sections three times in 0.1 M PBS before being incubated in 0.5% H_2_O_2_ in 0.1 M PBS for 30 min at room temperature to reduce endogenous peroxidase activity. Following another three 0.1 M PBS rinses, we incubated sections in 10% Normal Goat Serum (Vector Laboratories) in 0.3% Triton X-100 (Sigma) in 0.1 M PBS (PBS-T) for 60 min at room temperature. We then removed sections from the blocking serum into the primary Fos antibody (rabbit-anti-Fos antibody diluted 1:1000 in PBS-T, Santa Cruz Biotechnology K-25) and incubated for 21 h at room temperature. This antibody has previously been validated for use in the zebra finch (see Ref. [23]). The following day, we rinsed sections three times in 0.1% PBS-T and incubated in biotinylated goat anti-rabbit secondary antibody (diluted 1:250 in 0.3% PBS-T; Vector Laboratories) for 1 h at room temperature. After three rinses in 0.1% PBS-T, we incubated sections at room temperature in ABC Elite avidin-biotin horseradish-peroxidase complex (Vector Laboratories) for 1 h. Following three rinses in 0.1% PBS-T we visualized the antibody-avidin-biotin complexes with 0.04% diaminobenzidene solution (Sigma Fast DAB) for 90 s and then rinsed 4 times with 0.1 M PBS. We then serially mounted tissue sections on to Polysine microscope slides (VWR), serially dehydrated through alcohol (50–100%), cleared in xylene, and cover-slipped with DePeX (VWR). We found no immunoreactivity when we omitted the primary antibody.

### 2.7 Quantification of Fos immunoreactivity

In males, we quantified the number of nuclei expressing Fos in HVC and RA in the song-control system. We also quantified Fos immunoreactivity in LAI and DLN of the posterior motor pathway and AMV, AN, and ASt of the anterior motor pathway as identified in Feenders et al. [12]. In the social behavior network, we quantified Fos immunoreactivity in brain regions previously reported to increase immediate early gene expression with nest box possession in starlings: BSTmd, BSTmv, AH, POM, and VMH [17,20]. We also quantified Fos immunoreactivity in the social behavior network in two other divisions of the bed nucleus of the stria terminalis (BSTmv, BSTl), four divisions of the septum (LScv, LScvl, LSr, MS), and TnA as identified by Goodson [15] and Heimovics and Riters [17]. Because BSTmd and BSTmv have been found to both increase Fos immunoreactivity with nest box possession but are differentially influenced by breeding condition [17], we opted to sample these subdivisions separately, unlike a recent study testing for a role of vasotinergic neuron populations in BSTm in nesting [16]. We quantified Fos immunoreactivity in two regions of the hippocampus (dHP and mHP). In the dopaminergic reward/motivation circuit, we quantified Fos immunoreactivity in VTA and GCt.

**Fig. 1:**
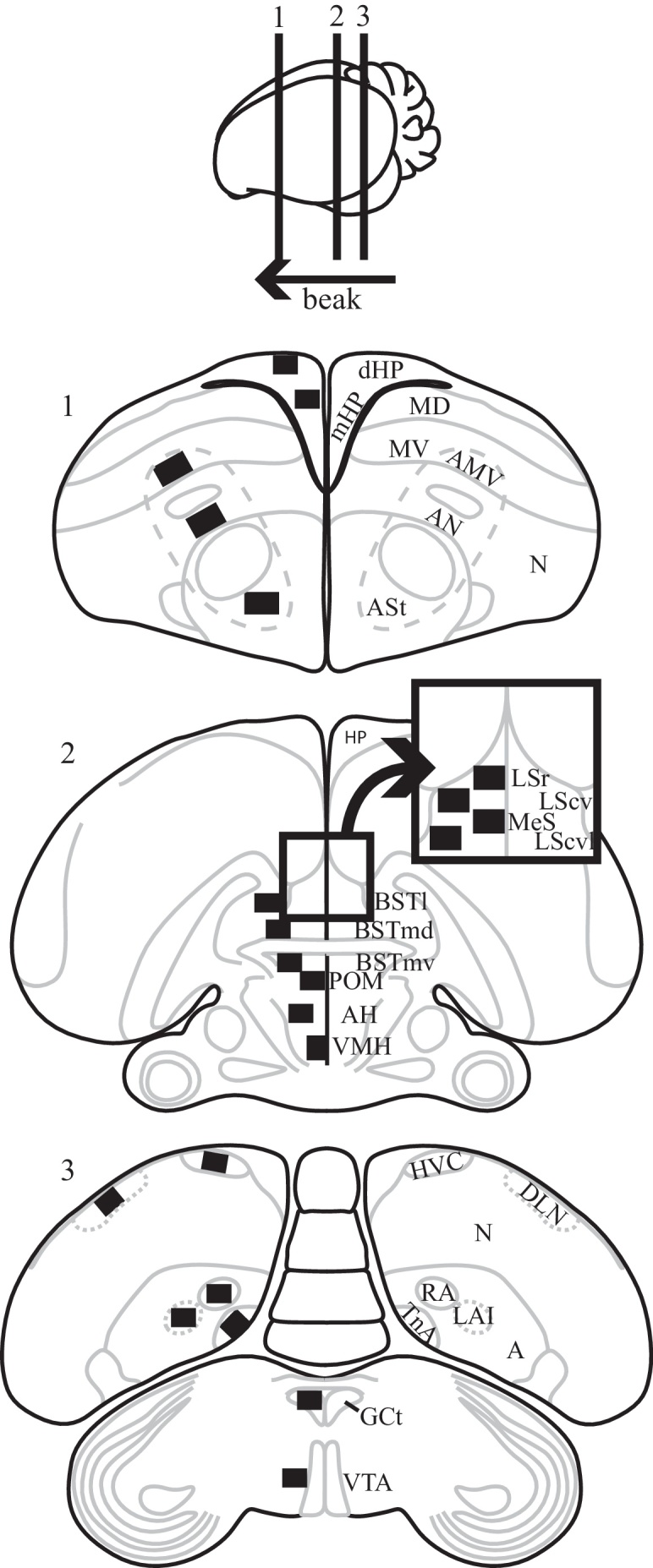
Brain regions quantified for Fos immunoreactivity in the zebra finch brain. Drawing of three transverse brain sections (1–3) and their locations along the sagittal plane (top diagram) depicting all regions quantified bilaterally for Fos immunoreactivity in this study. Black squares on the left hemisphere represent sampling squares taken at 40× objective magnification and brain region acronyms are located in the relative position of the sampling square in the right hemisphere.

We located areas of interest in brains using full section architecture and regional anatomy with reference to brain atlases of the canary [24] and zebra finch [25]. At each area of interest, we inspected adjacent coronal sections to locate the midpoint of the region in the rostrocaudal axis (Fig. 1). We took images of each region in both hemispheres and across 3 consecutive coronal sections centered on the rostrocaudal midpoint of the region (intersection interval = 90 μm). Regions larger in the rostrocaudal plane (ASt, dHP, and mHP) were quantified across 5 evenly-spaced coronal sections centered on the rostrocaudal midpoint of the region with an intersection interval of 270 μm. Images were taken using a Nikon Coolpix E4500 digital camera mounted on a Leitz Diaplan microscope using a 40× objective lens and Leitz Wetzlar 307-148.001 light source.

During quantification, each image was opened in ImageJ software (version 1.45, NIH, Bethesda, MD, USA) and desaturated. To isolate Fos nuclei from background staining, we used the *auto levels* function in ImageJ, which saturates a lack of Fos immunoreactivity as white and saturates Fos immunoreactivity as black. Before applying the function to each image, we subtracted 40 units from the *auto levels* adjustment value. An experimenter blind to bird treatment confirmed that this subtraction reliably highlighted darkly-stained Fos nuclei from background staining in a set of randomly selected images from multiple birds and brain regions. In the anterior motor pathway regions (ASt, AN, and AMV), only 30 units were subtracted from the *auto levels* value as the same experimenter (blind to bird treatment) found that neuropil staining was notably lighter and better excluded using this modified *levels* manipulation. After applying this function, the number of highlighted Fos immunoreactive nuclei were counted using the *analyze particles* function in ImageJ. Nuclei were counted if they had a minimum area of 400 pixels^2^. This value was selected by an experimenter blind to bird treatment by measuring the area of the smallest Fos immunoreactive nuclei identified in multiple, randomly-selected regions across birds and brain regions. The number of Fos immunoreactive nuclei in each hemisphere and section were summed to yield a single value for each brain region in each bird. Total Fos immunoreactive nuclei counts for each brain region were used in statistical analysis except for HVC as lateralization in activation in the right hemisphere has been previously reported during short-distance communication with a sexual partner in zebra finches [26]. In HVC, we analyzed Fos immunoreactivity in the left and right hemispheres separately.

### 2.8 Statistical analysis

During the behavioral analysis, one pair of nesting finches was identified as an outlier as the male picked up only small amounts of nest material (<2 SD below the mean for the rest of nesting males) and the female was never observed interacting with the nesting material within the nest cup. As a result we excluded this pair from further statistical analysis.

All statistical analyses were performed using PASW software (version 19.00, SPSS Inc., Chicago, IL, USA). We quantified finch behavior 80–50 min prior to sacrifice. The delay between quantified behavior and sacrifice provided sufficient time for the accumulation of Fos protein following neural activation associated with nesting [27]. All behavior and Fos data were normally distributed (*p* > 0.05; Shapiro–Wilk). We compared behavior and Fos immunoreactivity counts as dependent variables using GLMs and the independent variables included sex on two levels (male and female) and treatment on two levels (nesting and control). For the Fos data, we looked specifically for treatment and treatment x sex interaction effects that reflected neural activity associated with nesting.

To investigate whether nesting behaviors explain individual variation in Fos production, we entered all recorded behaviors in nesting birds as independent predictors of Fos immunoreactive nuclei quantified in each brain region using multiple linear regression. We ran regression models separately for males and females using a stepwise reduction procedure excluding interactions between types of behavior. In the song control nuclei (HVC and RA), we entered singing behavior (*song bouts* and *time spent singing*) as predictors of Fos immunoreactive nuclei counts in all males (nesting and control) firstly to test for song-brain correlations as previously reported [28] and secondly to test for a relationship between Fos immunoreactivity and the variation in the birds’ behavior.

## 3 Results

Regressional models in which nesting behavior significantly explained variation in Fos production in a brain region are summarized in Table 1.

### 3.1 Behavioral analyses

Between 80-50 min prior to sacrifice, control birds hopped (*F*_1,26_ = 22.623, *p* < 0.001), fed (*F*_1,26_ = 9.617, *p* = 0.005), drank (*F*_1,26_ = 7.296, *p* = 0.012) and preened (*F*_1,26_ = 6.049, *p* = 0.021) more than did nesting birds. Males scratched more often than did females (*F*_1,26_ = 20.362, *p* < 0.001).

Control females tended to allopreen more than did nesting females (*t*_13_ = 1.991, *p* = 0.087). Nesting and control males did not significantly differ in the time they spent singing (*p* > 0.05). In nesting pairs, males visited the nest cup more often than did females (*t*_12_ = 6.128, *p* < 0.001) but did not spend more time in the nest cup (*p* > 0.05).

Time spent singing was positively correlated with Fos immunoreactivity in the right (*β* = 0.692, *t* = 3.457, *p* = 0.004) but not in the left hemisphere (*p* > 0.05) in all males. Neither the number of song bouts nor time spent singing significantly explained variation in Fos expression in RA (all *p* > 0.05).

### 3.2 The motor pathways

**Fig. 2:**
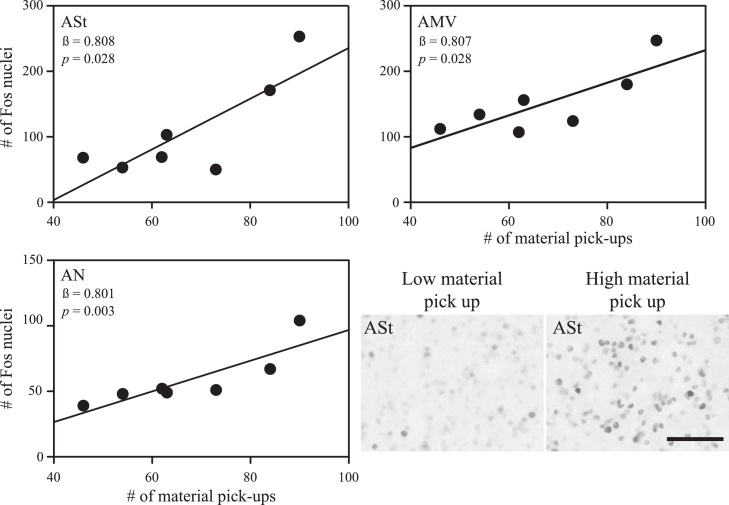
Correlations between nest-building behaviors and Fos immunoreactivity in the anterior motor pathway in zebra finches. Lines represent significant correlations between the picking up of nesting material and the number of Fos immunoreactive nuclei quantified in regions within the anterior motor pathway (*p* < 0.05) in adult male zebra finches. Correlations were derived from stepwise linear regressions. Within each graph, the regression coefficient and *p* value of the model are presented in the top left corner. Micrographs of sampling squares taken in tissue stained to label neurons producing Fos in ASt in the right hemisphere of a male finch who picked up the most and a male finch who picked up the least amount of material while constructing a nest (bottom right). Scale bar represents 50 μm. ASt = anterior striatum; AN = anterior nidopallium; AMV = anterior ventral mesopallium.

The number of times males picked up pieces of nesting material (Fig. 2; *β* = 0.808; *t* = 3.070; *p* = 0.028) was positively correlated with variation in Fos immunoreactivity in ASt. The number of times the males picked up material (Fig. 2; *β* = 0.801; *t* = 6.451; *p* = 0.003) and time spent singing (*β* = 0.459; *t* = 3.696; *p* = 0.021) were both positively correlated with the variation in Fos immunoreactivity in AN. The number of times the males picked up material (Fig. 2; *β* = 0.807; *t* = 3.061; *p* = 0.028) was positively correlated with variation in Fos immunoreactivity in AMV. Variation in nesting behaviors did not explain the variation in either of the areas we quantified from the posterior motor pathway, LAI or DLN (*p* > 0.05).

In nesting females, neither the number of visits to the nest nor the time spent in the nest significantly explained the variation in Fos immunoreactivity in either the anterior or posterior motor pathway (*p* > 0.05).

We also found no significant difference in Fos immunoreactivity between nesting and control birds in either the anterior or posterior motor pathway (*p* > 0.05).

### 3.3 The social behavior network

**Fig. 3:**
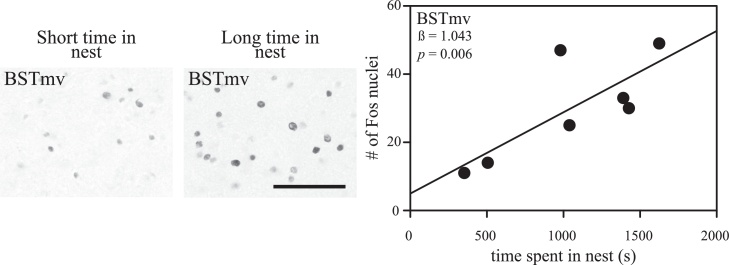
Correlations between nesting behaviors and Fos immunoreactivity in the social behavior network. Lines represent significant correlations between nesting behaviors (the depositing of nesting material in males and the time spent in the nest cup in females) and the number of Fos immunoreactive nuclei in bed nucleus of the stria terminalis, medioventral division (*p* < 0.05). Correlations were derived from stepwise linear regressions. Within each graph, the regression coefficient for the behavior and model *p* value are presented. Micrographs of sampling squares taken in tissue stained to label neurons producing Fos in BSTmv in the right hemisphere of a female finch who spent the most time in her nest and a female finch who spent the least amount of time in her nest (bottom left). Scale bar represents 50 μm.

The more pieces of material the males deposited in the nest cup the less Fos immunoreactivity we observed in MS (Fig. 3; *β* = −0.795; *t* = −2.928; *p* = 0.033). Fos immunoreactivity was higher in LScv and lower in VMH the more time nesting males spent singing (LScv: *β* = 0.928; *t* = 5.555; *p* = 0.003; VMH: *β* = −0.792; *t* = −2.899; *p* = 0.034). Fos immunoreactivity in LSr was lower the more times nesting males hopped (Fig. 3; *β* = −0.778; *t* = −2.771; *p* = 0.039) while neither picking up nor depositing nest material significantly explained variation in Fos immunoreactivity in any of the other social behavior network regions that we quantified (*p* > 0.05).

Fos immunoreactivity in AH decreased with increasing amount of time nesting females spent in the nest (Fig. 3; *β* = −0.771; *t* = −2.711; *p* = 0.042). Fos immunoreactivity in BSTmv, however, was higher the more time these females spent in the nest (Fig. 3; *β* = 1.043; *t* = 5.399; *p *= 0.006) and the more time they spent preening (*β* = 0.595; *t* = 3.079; *p* = 0.037). Fos immunoreactivity in VMH was higher the less time the nesting females spent preening (*β* = −0.861; *t* = −3.790; *p* = 0.013). Neither the number of times these females visited the nest nor the time these females spent in the nest significantly explained variation in Fos immunoreactivity in any other social behavior network regions quantified (*p* > 0.05).

Fos immunoreactivity in BSTmd (*F*_1,23_ = 4.720, *p* = 0.040) and POM (*F*_1,25_ = 8.095, *p* = 0.009) was significantly greater in nesting birds relative to control birds. There was no significant difference in Fos immunoreactivity between nesting and control birds in any other region sampled (*p* > 0.05).

### 3.4 The dopaminergic reward system

**Fig. 4:**
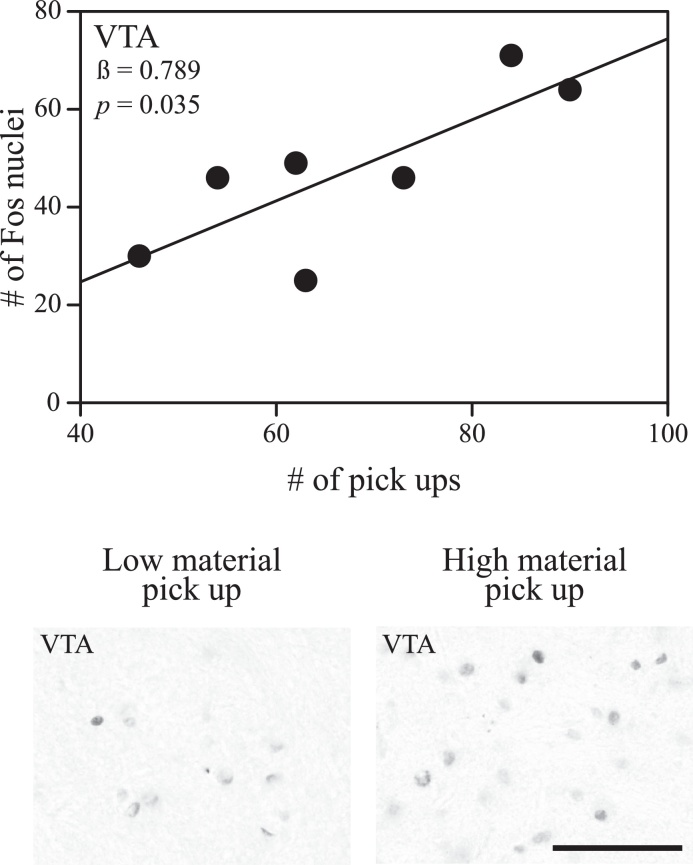
Correlations between nest-building behaviors and Fos immunoreactivity in the dopaminergic reward system. Lines represent significant correlations between the picking up of nesting material and the number of Fos immunoreactive nuclei quantified in the ventral tegmental area (*p* < 0.05) in adult male zebra finches. Correlations were derived from stepwise linear regressions. Within the graph, the regression coefficient for the behavior and model *p* value are presented. Micrographs of sampling squares taken in tissue stained to label neurons producing Fos in the ventral tegmental area in the right hemisphere of a male finch who picked up the most and a male finch who picked up the least amount of nesting material while constructing a nest (right). Scale bar represents 50 μm.

Fos immunoreactivity in VTA increased with the number of times the nesting males picked up pieces of nest material (Fig. 4; *β* = 0.789; *t* = 2.870; *p* = 0.035). Conversely, variation in nesting behavior did not significantly explain variation in Fos immunoreactivity in GCt (*p* > 0.05).

Neither the number of times the nest was visited nor the time spent in the nest by the nesting females significantly explained variation in Fos immunoreactivity in VTA or GCt (*p* > 0.05).

Fos immunoreactivity in VTA and GCt did not differ between nesting and control birds (*p* > 0.05).

### 3.5 Hippocampus

Nesting behavior and Fos immunoreactivity in dHP and mHP were not correlated (*p* > 0.05). We also found no significant differences in Fos immunoreactivity in dHP and mHP between nesting and control birds (*p* > 0.05).

## 4 Discussion

Using immediate early gene immunohistochemistry, we have identified regions of the songbird brain that produce Fos during nest building. This Fos production presumably is reflecting neural activation [11] within the anterior motor pathway, social behavior network, and dopaminergic reward system as Fos immunoreactivity was positively correlated with the number of times nest material was picked up by nest-building males or with the time spent in the nest cup by nesting females. This is the first demonstration of neural correlates of nest-building behavior in the anterior motor pathway and dopaminergic reward circuit.

### 4.1 Motor pathways

The number of times a male finch picked up nest material explained variation in Fos production throughout the anterior, but not posterior, motor pathway. Given the involvement of the anterior motor pathway in motor learning and sequencing [12], activation of the anterior motor pathway, and ASt in particular, during nest building suggests that nest-building behavior may involve similar motor control as has been ascribed to tool use behavior (which activates the basal ganglia in primates: [14]). Fos production in the anterior motor pathway was, however, specifically related to initiation of the sequence of nest-building behavior (picking up material) but not to the final step in the behavioral sequence that we were able to quantify (depositing material in the nest). This suggests that the AN in the zebra finch brain (as identified by [12]) is functionally similar to NIML in the pigeon brain (as identified by Ref. [29]), which plays a role in initiating learned motor sequences.

Conversely, the number of visits the females partnered to nest-building males made to the nest and time they spent in the nest cup were unrelated to Fos immunoreactivity in the anterior motor pathway. This sex difference suggests that, during nest building, the anterior motor pathway is specifically involved in the collection of nest material and not construction within the nest cup, in which both male and female zebra finches participate [9]. Our measures of nesting in female finches, however, were restricted to nest visitation and the time they spend in the nest may not reflect the degree to which they carry out any construction behavior within the nest cup. Collection of construction behavior data within the nest by both birds is required to specifically address (a) whether the anterior motor pathway might be involved in female nesting behavior and (b) whether it is involved in motor sequencing in males.

### 4.2 Social behavior network

There was significantly more Fos immunoreactivity in POM and BSTmd of nesting finches compared to control birds. In conjunction with previous reports of increased Fos production in POM and BSTmd during nest box possession in adult male starlings [17], our failure to find correlations between Fos immunoreactivity in POM and BSTmd and nest building suggest that this activity is associated with nest possession.

Although we did not find a group difference in Fos immunoreactivity in BSTmv, Fos production increased the longer the females spent in the nest. Elevation of Fos production in BSTmv following nest box possession has been attributed to concurrent changes in agonistic behavior associated with territorial defense of the nest [17]. Our results in female finches, however, suggest that such changes may be associated with occupation of the nest, a behavior that is only possible after a nest site has been obtained. Similar to Heimovics and Riters [17], we found that immediate early gene expression was higher in both BSTmd and BSTmv the more nesting behaviors the birds performed but those expression patterns differed. The differences in expression patterns dependent on the subdivisions of BSTm that are sampled may explain why there appeared to be no relationship in nesting birds between Fos production across the whole of BSTm and activation of vasotinergic neurons in BSTm [16].

### 4.3 Dopaminergic reward system

Fos immunoreactivity in VTA increased the more pieces of material the male finches picked up. As with the increase in Fos expression we observed in the BSTmd, the data suggest that Fos expression in VTA might be associated with nest building itself rather than with of other behavioral changes that occur after a nest site is obtained that are unrelated to nest building [17].

In addition to a potential role in reward during nest building, VTA may also influence activity in the anterior motor pathway during nest building. In vertebrates, VTA contains dopaminergic projection neurons. Studies in mammals have demonstrated that these neurons innervate the striatum and provide necessary dopamine to support basal ganglia functions including motor learning and sequencing [30,31]. This possibility of a role of the VTA on influencing activity of the anterior motor pathway is supported by our observation that Fos immunoreactivity was higher in both VTA and ASt the more nest material the males collected. Further examination of the relationship between Fos immunoreactivity in dopaminergic neuron populations in VTA and nest building is required to test this prediction.

### 4.4 Hippocampus

The absence of a correlation between variation in Fos expression in dHP and mHP and nesting behavior in male or female finches suggests that the hippocampus does not play a substantial role in nest building, at least in zebra finches.

### 4.5 Singing and HVC

Finally, we confirmed that Fos immunoreactivity is higher in the HVC as males spent more time singing. Furthermore, the time a male spent singing explained the variation in Fos expression better than did the number of song bouts [28,32].

## 5 Conclusion

Nest building in zebra finches involves the motor sequence of material collection and deposition by the male while the female visits the nest to receive material and shape the nest. Here we identified neural regions that varied in activity, as indicated by expression of the immediate early gene c-*fos* protein product Fos (the anterior motor pathway, social behavior network, and dopaminergic reward system), concomitantly with variation in nest building in male zebra finches and nesting in their mates. These are the first detailed data to show the neural underpinnings of construction behavior in birds (see also Ref. [33]) and are, therefore, a major step in determining the role that motor planning and sequencing, context recognition, and reward and motivation may play in those behaviors.

## Figures and Tables

**Table 1:** Nesting behavior correlates of Fos production in brain regions of adult zebra finches. Correlates were calculated using stepwise linear regression to identify behaviors performed by adult nesting zebra finches 50–80 min before sacrifice that predicted Fos production in sampled brain regions. When regression models included more than one behavior predicting Fos production in a single brain region, each behavior in the model is listed in the order of predictive power. Nesting behaviors are represented in bold.

Brain Region	Acronym	Sex	Correlated behavior(s)	β	*t*	*p*
Motor pathways						
Anterior striatum	ASt	Male	**Pick up**	0.808	3.070	0.028
Anterior nidopallium	AN	Male	**Pick up**	0.801	6.451	0.003
Anterior nidopallium	AN	Male	Time spent singing	0.459	3.696	0.021
Anterior ventral mesopallium	AMV	Male	**Pick up**	0.807	3.061	0.028
Social behavior network						
Anterior hypothalamus	AH	Female	**Time in nest**	−0.771	−2.711	0.042
Bed nucleus of the stria terminalis, ventromedial subdivision	BSTmv	Female	**Time in nest**	1.043	5.399	0.006
Bed nucleus of the stria terminalis, ventromedial subdivision	BSTmv	Female	Preening	0.595	3.079	0.037
Medial septum	MS	Male	**Put down**	−0.795	−2.928	0.033
Dopaminergic reward circuit						
Ventral tegmental area	VTA	Male	**Pick up**	0.789	2.870	0.035
